# Burnout Among Healthcare Providers in a Comprehensive Cancer Center in Saudi Arabia

**DOI:** 10.7759/cureus.3987

**Published:** 2019-01-30

**Authors:** Abdullah Bany Hamdan, Sami Alshammary, Sherwynn Javison, Jesusa Tamani, Musa AlHarbi

**Affiliations:** 1 Oncology, King Fahad Medical City Comprehensive Cancer Center, Riyadh, SAU; 2 Palliative Care, King Fahad Medical City Comprehensive Cancer Center, Riyadh, SAU

**Keywords:** burnout, cancer, strategies, distress, exhaustion, healthcare, oncology healthcare professionals

## Abstract

Background

Overwork has grave consequences for staff health, either physically or psychologically. Burnout has an impact on health care turnover, patient safety, patient satisfaction, and patient perception towards health professionals. This study aims to assess the prevalence of burnout, psychosocial distress, occupational predictors, perceived causes, and suggested strategies for preventing or reducing its impact of burnout on oncology healthcare workers.

Materials and methods

A cross-sectional survey was conducted among various oncology healthcare professionals using the Maslach Burnout and Kessler-10 Inventory tools to derive the data.

Results

A total of 157 participants represented with an overall response rate of 62.8%. Among all the respondents, it showed that 28.7% of them reported moderate to severe burnout. Moreover, 32.9% of the participants with patient contact had experienced moderate to severe burnout, and the same burnout level was reported by 55% of the respondents with no patient contact. Physicians (35.1%) were recorded to have the highest rate of burnout, followed by nurses (29%) and allied healthcare professionals (27%). Also, exhaustion and emotional exhaustion subscales were higher to those samples without patient contact (33.3%) compared to samples with patient contact (25.5%). On the other hand, 28.7% of those samples with patient contact exhibited a high level of depersonalization, while 42.9% of non-patient contact samples recorded a high level of cynicism. Both sub-samples scored more than half in personal accomplishment (73.4%) and the related professional efficacy (57%), merging the average and high-level scores. The proportion of non-patient contact respondents who had experienced psychiatric symptoms was 10%.

Conclusions

There was a significant number of King Fahad Medical City Comprehensive Cancer Center healthcare professionals who experienced a substantial level of burnout. On the other hand, the respondents listed different strategies to reduce the level of burnout. These strategies are self-defined, such as improved access to leave, attention to staff psychosocial and training needs, and emphasizing the importance of regular communication skills training. The management needs to take action for the area of improvement based on the results.

## Introduction

Modern cancer management offers intricate treatment strategies that enable highly favourable diagnostic and treatment options. However, advancement in cancer management has created a direct impact on healthcare professionals caring for these patients [1]. Burnout is a sign of emotional exhaustion, depersonalization, and a sense of low personal accomplishment which affect work productivity [2-3]. Work environment with factors such as deficient communication skills proficiency, low job controls, and high workloads are among those frequently reported as significant predictors of burnout. Furthermore, work and family life interference have been linked to predicting job stress [4]. Frequent time demands of the family may influence the performance and productivity of healthcare providers at work [5].

It is perceived to be extremely stressful to be working with cancer patients, particularly those diagnosed with an advanced metastatic state [6]. The work-related stress has a direct implication on psychological and physical aspects of the oncology staff. This severe concern should not be taken for granted as it will result in the deterioration of the personal and professional life of healthcare employees [7]. A broad range of healthcare professions, including physicians, nurses, and other allied healthcare professionals in the Comprehensive Cancer Center, may experience burnout.

Shortage of staff is one of the causes of burnout due to work overload, working full-time, and working overtime. These factors are most associated with job dissatisfaction and might lead to searching for another organization to work for [8].

Some examples of work experiences already encountered by healthcare professionals that resulted in the development of burnout are frequent patient complaints of physical pain, ethical dilemmas about treatment decisions, the intensive and complex nature of therapy, and managing professional boundaries [4, 9-10].

Communication skills proficiency is also a burnout factor for healthcare professionals [1, 11]. This kind of burnout was linked to emotional job demands termed as the emotional contagion that affects oncology professionals' care abilities towards confrontation with death and dying patients [12]. Therefore, professional burnout has a severe and direct effect on patient safety.

Providing opportunities and time to cope with burnout efficiently is beneficial to healthcare professionals’ health and well-being as it will decrease the prevalence of exhaustion and psychological distress [4]. Oncology staff that experienced burnout need to regain energy, replenish the strength to face existing problems, and to have time for self-devotion or self-reflection [2]. Also, enhancing the coping mechanism of oncology and palliative care physicians is essential as they encounter more challenges in the workplace [13]. An environment that supports staff to develop positive attributes benefits the organization by supporting work longevity and quality of patient care [4, 9]. The aim of our research study was to examine the occurrence of burnout, assess the level of burnout according to healthcare professionals’ job, identify significant causes, and recognize personal strategies for preventing and/or reducing burnout among various healthcare professionals working in the Comprehensive Cancer Center of King Fahad Medical City.

## Materials and methods

Study design

A cross-sectional survey was designed and used at Comprehensive Cancer Center in King Fahad Medical City in Riyadh, Kingdom of Saudi Arabia between June 2016 - June 2017 through an online survey questionnaire sent via email to all participants for completion of a self-assessment survey.

Study participants

The study population consisted of all healthcare professionals of the Comprehensive Cancer Center in King Fahad Medical City involving nurses, physicians, and other multidisciplinary team members working at the time of the survey. However, staff members that were on leave, not willing to participate, or not working in the field of cancer care were excluded in the study.

Procedure and ethical considerations

An email was sent to all staff members explaining the aim of the study, nature, the potential risk, and benefits. Prior to completing the online questionnaire, a web link would ask for the staff’s agreed participation or consent. Upon completion of the survey, a thank you email receipt was received by the participants. The confidentiality and anonymity of the participants were maintained throughout the study process. The King Fahad Medical City Institutional Review Board approved the study (IRB number: 15-156), and there was no conflict of interest identified.

Research instruments

An English language version of the validation tools was adapted from previous related studies with the permission of earlier researchers. A pilot study was performed with a minimum number of healthcare professionals in the same location. The final questionnaire was categorized into demographics, identifying the work factors, perception of burnout using the Maslach Burnout Inventory (MBI), assessment of the psychological distress using the Kessler-10 (K-10) and the psychiatric morbidity using the General Health Questionnaire-12 (GHQ-12) scales, enlisting burnout causes and preventive strategies in their own perception, and identifying communication skills training participation.

Measures and statistical analyses

A descriptive analysis was made using means and percentages to analyze the demographic profiles of the participants, burnout occurrence, and psychiatric morbidity. Data analysis was conducted using the IBM Statistical Package for Social Sciences (SPSS), vs. 22.0 (IBM SPSS Statistics, Armonk, NY).

Demographic Characteristics

Participants were asked a set of questions about their social demographic profile, such as gender, age, educational level, occupation type, working hours, patient contact type, and leave arrangement, respectively.

Burnout Prevalence

Participants were asked to answer the Maslach Burnout Inventory (MBI) questionnaire based on their patient contact type. The MBI Human Service Survey (HSS) is a tool used for respondents with patient contact which consists of 22 questions which are measured using the 7-point Likert scale. This tool measures the three subcategories, such as emotional exhaustion (feeling of emotionally overextended and exhausted in work), depersonalization (measures an unfeeling and impersonal response toward patients in one's care), and personal accomplishment (feeling of competence and achievements in one’s work with people). On the other hand, the MBI General Survey (GS) tool was used for the non-patient contact respondents which consisted of 16 questions and was measured using the 7-point Likert scale focusing the three subcategories that included exhaustion (measures exhaustion in connection to one's job without relating to emotions or social), cynicism (feelings of indifference or distant attitude towards work), and professional efficacy (sense of competence and successful achievements in one’s job with specific focus on expectations of continued effectiveness at work) [1]. Burnout was identified based on the cut-off points recommended by the MBI tools.

Psychological Condition

All the respondents answered the Kessler-10 (K-10) tool to assess their psychological condition or feelings over the past four weeks. The questions were measured using the 5-point Likert scale ranging from "none of the time” up to “all of the time." Analyses of the data interpreted the psychological distress level from low (10-19), moderate (20-24), high (25-29), and very high (30-50) [14].

Psychiatric Morbidity Condition

Non-patient contact samples were redirected to answer the 12-item General Health Questionnaire (GHQ-12) tool to measure the occurrence of psychiatric symptoms. This tool only intends to know the significant deviation of healthy psychological functioning. Each question was graded using the 4-point Likert scale, such as "not at all" (1) to "much more than usual" (4); however, for this study, the Likert scale scoring system of 1-2-3-4 was converted to 0-0-1-1 scoring system to eliminate possible bias. Affirmative question numbers 1, 3, 4, 7, and 12 were scored inversely [15]. The total scores were summed with the resulting score of 0 to 12. The cut-off point of 6 was based on overall GHQ scores because of various threshold cut-offs [16].

Burnout Causes and Strategies

The respondents who had burnout symptoms were then asked to state the burnout causes, preventive strategies, and communication training. 

## Results

A total number of 157 out of 257 eligible respondents participated in the study and fully completed the questionnaire, providing an overall response rate of 62.8%; 62 (39.5%) were males and 95 (60.5%) were females with a mean age of 35 years. Most of them had a university degree (46.5%). Nurses comprised over half of the sample (54.1%), and the remaining 35.1% were physicians and 10.8% were among allied healthcare professionals. Half of the respondents had worked an average of five years in the area of cancer care (52.8%). The majority had direct patient interaction (87.3%), and most of them worked for 40 - 45 hours/week in paid employment (95.6%). Meanwhile, most of the respondents engaged in a different level of unpaid work as part of their job at less than eight hours (98.7%), and 39.5% of them were not satisfied with their current leave arrangements (Table 1).

**Table 1:** Demographic Characteristics

Variables	Frequency n (%)
Gender	
Male	62 (39.5)
Female	95 (60.5)
Age	
20-29	49 (31.2)
30-39	66 (42.0)
40-49	38 (24.2)
> 50	4 (2.6)
Education	
Certificate/Diploma	27 (17.2)
Hospital Training/College Diploma	05 (03.2)
University Degree	73 (46.5)
Higher Degree (postgraduate)	45 (34.4)
Others	07 (04.5)
Occupation	
Nurse	85 (54.1)
Physicians	55 (35.1)
Allied Health	17 (10.8)
Years in Occupation	
0-5	50 (31.8)
6-10	69 (43.9)
11-15	24 (15.3)
16-20	08 (05.1)
> 20	06 (03.8)
Years in Cancer Center	
0-5	83 (52.8)
6-10	61 (38.9)
11-15	08 (05.1)
16-20	03 (01.9)
> 20	02 (01.3)
Paid Hours of Work	
<8 hrs./day	78 (49.7)
= 8 hrs./day	72 (45.9)
>8 hrs./day	07 (04.5)
Unpaid Hours of Work	
< 8 hrs./day	155 (98.7)
> 8 hrs./day	02 (01.3)
Patient Contact	
Yes	137 (87.3)
No	20 (12.7)
Satisfaction with the Current Leave Arrangement	
Not at all/not very satisfied	62 (39.5)
Somewhat satisfied	46 (29.3)
Quite/very satisfied	49 (31.2)

The prevalence of self-defined burnout was 28.7% in the moderate to severe level. The results revealed 32.9% (patient contact) and 55% (non-patient contact) of the respondents had experienced moderate to severe burnout (Table 2). The professional group with the highest prevalence of burnout were physicians (44%), followed by nurses (29%), and other health professionals (27%), as shown in Table 3.

**Table 2:** Prevalence of Self-defined Burnout in the Respondents with Patient Contact

	Burnout N (%)			
	NO n/%	LOW n/%	MODERATE n/%	SEVERE n/%
Total Sample (n = 157)	37 (23.5)	75 (47.8)	34 (21.7)	11 (7)
Patient Contact				
Yes (n = 137)	27 (19.7)	65 (47.4)	29 (21.2)	16 (11.7)
No (n = 20)	06 (30)	03 (15)	04 (20)	07 (35)

**Table 3:** The Percentage of Burnout in Each Job Category

Job Category	%
Nurses	29%
Physicians	44%
Allied Health	27%

Exhaustion and emotional exhaustion subscales were similar to those measured by the self-defined burnout scale. An average to high burnout level was noted in the respondents with patient contact (76.6%) compared to those with a single-item burnout level (23.4%). In addition, 28.7% of those respondents with patient contact exhibited a high level of depersonalization. Furthermore, 42.8% of those respondents with no direct patient contact scored high on cynicism. Both sub-samples scored more than half in personal accomplishment (73.4%) and the related professional efficacy (57%) merging average and high-level scores. Tables 4-5 are the results of the mean scores (M) and standard deviations (SD) for each of the three subscales using the two Maslach Burnout Inventories, the MBI-HSS, and MBI-GS.

**Table 4:** Prevalence of Burnout in Those with Patient Contact as Measured by the MBI-HSS ^a ^High scores indicate higher levels of burnout; ^b^ Low scores indicate higher levels of burnout M: mean; MBI-HSS: Maslach Burnout Inventory Human Service Survey; n: number; SD: standard deviation

	Emotional Exhaustion ^a^ (n = 94) M = 36.03, SD = 12.18 n/%	Depersonalization ^a^ (n = 94) M = 20.11, SD = 7.96 n/%	Personal Accomplishment ^b^ (n = 94) M = 27.2, SD = 11.0 n/%
High	24 (25.5)	27 (28.7)	24 (25.6)
Average	48 (51.1)	40 (42.6)	45 (47.8)
Low	22 (23.4)	27 (28.7)	25 (26.6)

**Table 5:** Prevalence of Burnout in Those Without Patient Contact as Measured by MBI-GS ^a ^High scores indicate higher levels of burnout; ^b ^Low scores indicate higher levels of burnout M: mean; MBI-HSS: Maslach Burnout Inventory Human Service Survey; n: number; SD: standard deviation

	Exhaustion ^a^ (n = 03 ) M = 12.6, SD = 03.09 n	Cynicism ^a^ (n = 07 ) M= 10.14 SD=7.56 n	Professional Efficacy ^b^ (n = 07 ) M = 18.4, SD = 5.2 n
High	01	03	03
Average	00	02	01
Low	02	02	03

Thirty-seven percent of the respondents had experienced a psychological disturbance in the previous few weeks from high to a very high level, while 25% experienced moderate levels and 38% experienced low levels as described in Table 6. Meanwhile, the proportion of the respondents who had experienced psychiatric symptoms was 10% in comparison to the general Saudi population (16%) [17]. There were three identified GHQ-12 factors with its corresponding values for mean (M) and standard deviation (SD): psychological distress (M = 0.24, SD = 0.3564), social and emotional dysfunction (M = 0.15, SD = 0.362), and cognitive disorder (M = 0.2, SD = 0.41), as shown in Table 7.

**Table 6:** Psychological Analysis Using Kessler Psychological Distress (K-10) Scale

Psychiatric Morbidity Level	Score Range	n	%
Low	10 - 19	59	38%
Moderate	20 - 24	39	25%
High	25 - 29	32	20%
Very High	30 - 50	27	17%

**Table 7:** Psychiatric Morbidity Analyses Using the General Health Questionnaire (GHQ-12) Scale M: mean; SD: standard deviation

GHQ-12 Psychiatric Symptom Occurrence	n	%
Cases	2	10%
Non-cases	18	90%
GHQ-12 evaluation based on three factors	M	SD
Psychological distress	0.24	0.3564
Social and emotional dysfunction	0.15	0.362
Cognitive disorder	0.2	0.41

The analysis of the perceived causes of burnout showed that 42.2% indicated the work environment as the dominant cause of burnout. Also, there were some contributing factors concerned with organizational issues. Almost one-third of the respondents expressed frustration with management as being non-responsive. Moreover, there was another familiar source of dissatisfaction, such as lacking understanding of job requirements, bureaucratic red tape, and funding constraints, mentioned respectively. Work-related burnout centered on issues of workload, working hours, financial matters, and access to leave. There were some who gave more indicators for this concern, and most of these were in line with the clinical work. On the contrary, 13.3% of the respondents mentioned that the workload as being excessive for the managerial and administrative tasks.

Staffing issues were about frustration at not being able to perform their work due to their workload (15.6%). There was no hiring and delay in staff hiring when they finished their contracts. Long working hours was considered by some of the respondents to be a significant factor that contributed to burnout but more importantly was the impact on the personal lives of health care providers. It means mind-set necessitates them to take work home to get the job done, having meetings after work, etc. Table 8 shows the breakdown of the respondent's comments about the perceived causes of burnout.

**Table 8:** Comments About Perceived Causes of Burnout for the Respondents Experiencing Moderate to High Level of Burnout

Perceived Causes of Burnout	n	%
Work-related financial causes	6	13.3%
Leadership style	13	28.9 %
Staffing related	7	15.6%
Work environment	19	42.2%

The results revealed that 62.8% reported encouraging positive atmosphere and organized the work process are essential strategies to prevent burnout, such as improving leave arrangements, more flexible work arrangements, justifiable clinical workload, and better access to professional development; 14.05% reported the need to recruit more staff would help to better manage the service and decrease the load of duties which could reduce burnout. In addition, some respondents (7.44%) perceived that access to professional development is a significant factor in the prevention of burnout which includes further study (e.g., postgraduate courses), research, professional development (e.g., continuous professional development, conference attendance), and staff education (e.g., job training). On the other hand, 9.09% talked about financial support that mostly included leave allowances and financial assistance (Table 9). Finally, almost half of the respondents had received some communication skills training within the previous three years, with local hospitals (37.8%) being the dominant provider. However, 39.4% reported a moderate and high need for further communication skills training, with 29% of them needing training in addressing patients' emotional requirements as shown in Table 10.

**Table 9:** Respondents’ Comments About Strategies in Preventing Burnout

Avoiding Burnout	n	%
Best resources utilization	7	5.78%
Create a friendly environment	1	0.83 %
Educational/training needs	9	7.44%
Encourage a positive atmosphere	51	42.15%
Financial support	11	9.09%
Organize the work	25	20.66%
Recruit more staff	17	14.05%

**Table 10:** Communication Skills Training (CST) Issues Reported by the Respondents with Direct Patient Contact * One respondent can select multiple options

Recent Communication Skills Training Received	n %
Within the last year	39 (28.5)
More than one but less than three years ago	29 (21.2)
More than three years ago	31 (22.6)
Never	38 (27.7)
Sources of Communication Skills Training	n %
Local hospital	31 (37.8)
Cancer Council	12 (14.6)
University postgraduate course	13 (15.9)
National Breast Cancer Center	04 (04.9)
Other	10 (12.2)
University undergraduate course	05 (06.1)
Private/external agency	02 (02.4)
Cancer Institute	02 (02.4)
Palliative care	03 (03.6)
Current Need for Communication Skills Training	n %
No need	25 (18.2)
Some need	58 (42.3)
Moderate need	30 (21.9)
High need	24 (17.5)
Areas of Communication Skills Training Needs *	n %
Addressing emotional issues	50 (29%)
Discussing sexuality issues	06 (4%)
Breaking bad news	26 (15%)
Discussing prognosis	14 (8%)
Discussing palliative care	24 (14%)
Discussing treatment options	28 (16%)
Considering clinical trials	17 (10%)
Interprofessional communication and conflict management	02 (1%)
Other	02 (1%)
Dealing with grief, death, and dying	01 (1%)

## Discussion

This study offered a unique opportunity to examine healthcare professionals’ perception of burnout experiences. The work factors are directly significant to healthcare professionals, particularly towards the issue of leave plans/arrangements which was linked to causing major burnout. Moreover, burnout incidence was identified among those engaged in less than eight hours of unpaid work. Fortunately, the findings suggest that a higher reduction of burnout can be achieved when there is an increased frequency of vacation leaves [3]. Personal demands of directly working with cancer patients have also been linked as a source of burnout for other respondents [6-7]. The empathetic manner of care to the sick and dying patients leads to overwhelming demands for personal resources, joined with the inability to have their grief dealt with appropriately. Furthermore, the study outcomes show non-patient contact respondents experience average to severe burnout levels compared to the respondents with the direct patient care that had only a low burnout level. Moreover, the results were consistent across self-defined burnout in which the degree is slightly higher among oncology and palliative care physicians compared to other healthcare professionals [18]. Healthcare providers with greater time spent in direct patient contact have experienced average to high emotional exhaustion levels. However, tangible benefits from a moderate patient load and increased personal accomplishment are evidence of rewards gained from patient contact.

Causes of burnout listed by direct patient contact samples offered an individualized comprehensive understanding of burnout. The overall result shows significant causes, such as work environment, leadership style, work-related financial reasons, staffing related, and insufficient vacation time. The proportion of non-patient contact respondents who experienced psychiatric symptoms was only 10%.

Another predictor which had a direct effect on high-level burnout was the communication skills training needs. The study generated significant findings that almost half of the respondents reported a moderate to high-level need for further communication skills training, and some of them stated nothing at all. The importance of staff participation in regular training and education is supported by findings that enhance staff competence and personal accomplishments. There is a definite link between communication skills training and individual achievement with direct patient contact [19].

The study results revealed significant personal preventative strategies, such as encouraging a positive atmosphere, organize work, recruit more staff, and financial support, respectively. Conducive and positive work environment enables healthcare professionals to do their job more efficiently without anything and anyone that bothers them [2].

## Conclusions

The results of the study reported that oncology healthcare professionals experienced a significant level of burnout. The healthcare organizations should recognize employee stress and exhaustion in the work environment. The organization may consider the suggested strategies in creating a personalized and sustainable approach to reduce burnout recurrence. The management may communicate with the stakeholders the need to improve staff work performance which leads to the improvement of patient outcome and synching patient safety thereafter.
